# Mid- and long-term clinical outcomes of corrective fusion surgery which did not acquired sufficient PI-LL value for adult spinal deformity

**DOI:** 10.1186/1748-7161-10-S1-P24

**Published:** 2015-01-19

**Authors:** Kentaro Yamada, Yuichiro Abe, Yasushi Yanagibashi, Takahiko Hyakumachi, Shigenobu Sato

**Affiliations:** 1 Wajokai Eniwa Hospital, Japan

## Objective

Recent studies have demonstrated sagittal spinal balance was more important than coronal balance in terms of clinical result of surgery for adult spinal deformity. Notably, Schwab reported one of the target spinopelvic parameters for corrective surgery was that lumbar lordosis (LL) should be within +/- 10 degrees of pelvic incidence (PI). The present study was aimed to investigate whether the clinical outcome of corrective fusion surgery was really poor for patients who could not be acquired sufficient PI-LL value by the surgery.

## Materials and methods

The present study included 13 patients who underwent corrective fusion surgery more than 4 intervertebral levels, were showed PI-LL more than 10 degrees on the standing X-ray immediately after surgery, and were followed up more than 3 years. Parameters using SRS-Schwab classification were investigated using the total standing spinal X-ray before, immediately after surgery, and at final follow-up. Proximal junctional kyphosis of more than 15 degrees, loosening of implants, and non-union were evaluated at final follow-up. Clinical outcomes were evaluated by Japanese Orthopaedic Association scores (JOA score), Oswestry Disability Index (ODI), SF-36, Visual Analog Scale (VAS) for low back pain, and satisfaction for surgery using SRS-22 questionnaire.

## Results

All patients showed the PI-LL more than 20 degrees before surgery. All surgeries were undergone by posterior approach. The fusion number was mean 6.0, PLIF number was mean 2.8. Five pedicle-subtraction-osteotomies was combined. Although the LL were acquired mean 23.6 degrees by surgery, significant loss of correction (mean 10.8 degrees) was observed at final follow up. The coronal spinal balances were maintained good balance within follow-up period. However, sagittal vertical axis (SVA) as sagittal spinal balance were significantly shifted forward from mean 4.5cm immediately after surgery to 11.1cm at final follow-up. Five patients showed PJK, 10 patients showed loosening of implants, 8 patients showed non-union at final follow-up. The JOA score and mental health summary measures of SF-36 were significantly improved at final follow-up. The other clinical outcomes were improved but not significant. The satisfaction score was mean 3.3 points, including 3 patients with over 4 points, at final follow-up. The satisfaction score correlated negatively with SVA at final follow-up.

## Conclusions

The forward shift of SVA was frequently observed, and SVA at final follow-up related patient's satisfaction of surgery. This study indicated the importance of postoperative PI-LL value, but also noted 23% of patients acquired good SVA and satisfaction unless the surgery acquired adequate LL.

